# Antioxidant and stress-adaptive properties of putative probiotic bacteria in Pakistani fermented buffalo milk

**DOI:** 10.3389/fnut.2025.1619212

**Published:** 2025-08-25

**Authors:** Gulnaz Saleem, Bisma Rao, Gul Bahar Khaskheli, Hengxian Qu, Md Shabuddin Ahamed, Muhammad Qasim, Ruixia Gu, Xia Chen

**Affiliations:** ^1^College of Food Science and Technology, Yangzhou University, Yangzhou, Jiangsu, China; ^2^Key Lab of Dairy Biotechnology and Safety Control, Yangzhou, Jiangsu, China; ^3^Department of Public Health, Medical College, Yangzhou University, Yangzhou, Jiangsu, China; ^4^Department of Animals Products Technology, Sindh Agriculture University Tandojam, Tando Jam, Pakistan; ^5^College of Food Science and Technology, Huazhong Agricultural University, Wuhan, China; ^6^Microelement Research Center, College of Resources and Environment, Huazhong Agricultural University, Wuhan, Hubei, China

**Keywords:** buffalo milk, fermented products, probiotic bacteria, stress tolerance, antioxidant activity

## Abstract

**Introduction:**

Fermented buffalo milk products from South Asia remain an underexplored source of microbial diversity with potential health-promoting benefits. This study investigates the probiotic and industrial suitability of lactic acid bacteria (LAB) and non-LAB isolates from traditional Pakistani dairy, addressing gaps in region-specific probiotic discovery.

**Methods:**

Forty-seven bacterial isolates were obtained from fermented buffalo milk products (yogurt and cheese). Molecular identification (16S rRNA sequencing) classified isolates into LAB and non-LAB taxa. Probiotic potential was evaluated via *in vitro* assays for gastrointestinal stress tolerance (pH 2.0, 0.5% bile), antioxidant activity (DPPH scavenging), and industrial adaptability (growth at 4–45°C, 2–6% NaCl).

**Results:**

Eight strains were prioritized, including *Lactobacillus plantarum* Y_1_, *L. brevis* Cc_3_, *Streptococcus thermophilus* Y_6_/Cc_1_/Cm_5_, and non-LAB *Bacillus dendritiformis* Y_9_. *L. plantarum* Y_1_ exhibited exceptional acid resistance (>5.0 log10 CFU/mL at pH 2.0) and bile tolerance (6.5 log10 CFU/mL). *L. brevis* Cc_3_ combined high bile resilience (6.0 log10 CFU/mL) with robust antioxidant activity (52% DPPH scavenging), while *S. thermophilus* Y_6_ showed 48% antioxidant capacity. Non-LAB isolates, particularly *B. dendritiformis* Y_9_, demonstrated unexpected bile stress survival (5.4–5.5 log10 CFU/mL). All strains grew under industrial conditions (4–45°C, 2–6% NaCl), except *S. thermophilus* Cc_1_, which was heat-sensitive above 40°C.

**Conclusion:**

This study highlights South Asian buffalo milk as a reservoir of both conventional LAB and novel non-LAB strains with dual stress tolerance and antioxidant functionality. *L. plantarum* Y_1_ and *L. brevis* Cc_3_ emerge as prime candidates for developing culturally tailored functional foods to address regional nutritional challenges. The resilience of non-traditional isolates such as *B. dendritiformis* Y_9_ challenges existing probiotic taxonomical biases, suggesting broader microbial resources for gut-health innovations. These findings advocate for integrating regionally adapted probiotics into functional diets to enhance gastrointestinal health and oxidative stress mitigation in South Asian populations.

## 1 Introduction

Lactic acid bacteria (LAB) comprise a diverse group of Gram-positive, non-spore-forming, catalase-negative, and aerotolerant bacterial species that are commonly known for their rods or cocci-like shapes (1). They commonly reside in nutrient-rich environments, such as vegetables, meat, milk products, and various drinks. Even now, their primary function is as starter cultures in the food sector, where they have been fermenting foodstuffs and animal feed for ages (2). They are the essential key players in the production of dairy food products. According to a study (3, 4), they have a reputation for being able to ferment foods, which makes them healthier, improves their organoleptic qualities, and increases the abundance of nutrients. Another common use of LAB and *Bifidobacterium* is as a probiotic. Research on the potential health benefits of probiotics containing LAB, such as *Lactobacillus* and *Pediococcus* spp., has demonstrated their roles in gut microbiota modulation, immune enhancement, and pathogen inhibition (5). Probiotics are classified as a common category in the class of famous food supplements. These are functional foods due to their greater health advantages than conventional nutritional products (6). In the interim, numerous scientific studies have been conducted in recent decades to select LABs with distinct and specific functional properties as well as novel probiotic bacteria that are continuously isolated and identified (7).

Due to the rapid growth of the probiotic industry, there remains a strong demand for probiotics. However, potential probiotic strains must first demonstrate resilience against the harsh conditions imposed by the human body (8). Oxidative stress, a key contributor to many disorders, occurs when the balance between the body's antioxidant defenses and free radical production is disrupted (9). LABs are beneficial because they regulate the balance of the intestine, lower the blood cholesterol, reduce the risk of cancer, and revitalize the immune system, among other things (10). They have anti-oxidative effects as reported by (11) and many previously reported studies, e.g., bacterial strains, such as *L. acidophilus, L. fermentum*, and *L. sake* (12–14). Additionally, *B. clausii* has been used and proven effective in treating diarrhea in humans. These agents have demonstrated significant immune-modulatory properties in numerous *in vitro* and clinical studies (11, 15).

In the past 20 years, global milk production has nearly doubled, with buffalo milk experiencing an annual growth rate of approximately 2.5% higher than cow milk (16). India, Pakistan, and China are the largest producers of buffalo milk, with Italy being the highest producer in Europe and ranking sixth globally (17). In numerous developing countries, buffalo milk plays a crucial role in meeting the nutritional needs of humans (16). Buffalo milk is an appropriate raw ingredient for the production of several types of fermented dairy products, including cheeses and artisanal cheeses. Because its components are richer than those of cow milk, it is ideal for processing a broad range of dairy products that are liked by many cultures as their traditional food (18). Since limited studies have been done to explore the probiotic potential of Pakistani buffalo milk, this study is an attempt to fill the existing gap. Moreover, given the rising global demands for region-specific probiotics, this study highlights the Pakistani fermented buffalo milk as an under-explored source of stress-resilient and antioxidative microbes. By characterizing both traditional and non-conventional isolates, we focused to identify the candidates with dual probiotic-industrial potential, offering novel solutions for functional foods tailored to South Asian nutritional and oxidative stress challenges. This research set out to conclude the variety of beneficial microorganisms present in fermented buffalo milk products.

## 2 Materials and methods

### 2.1 Fermented milk samples collection and transportation

A total of 20 samples (*n* = 20) of buffalo fermented milk products were collected under hygienic conditions from the local dairy farm/shops of the district of Hyderabad, Pakistan. At the time of collection, all the samples were labeled properly, mentioned in Table 1, and brought under chilled conditions (4 ± 1°C) to the Department of Animal Products Technology, Sindh Agriculture University, Tandojam, and CVD Laboratory Tandojam, Pakistan. Bacterial isolation was done by growing them on de Man, Rogosa, and Sharpe (MRS) agar medium and subculturing to get pure cultures.

**Table 1:** Sample collection details of fermented milk products.

**Sample type**	**Sample codes**	**Quantity**	**Container type**	**Collection site**	**Transport condition**
Yogurt	Y_1_-Y_10_	10	Sterile PP	Local dairy farms/shops	4 ± 1°C
Mozzarella cheese	Cm_1_-Cm_5_	5	Sterile PP	Local dairy farms/shops	4 ± 1°C
Cheddar cheese	Cc_1_-Cc_5_	5	Sterile	Local dairy farms/shops	4 ± 1°C

PP, Polypropylene containers.

### 2.2 Isolation of lactic acid bacteria from fermented milk

Initially, 1 mL of fermented milk was mixed with 9 mL of sterile distilled water (DW) to create a 10^−1^ dilution. Serial dilutions were then prepared to achieve appropriate bacterial concentrations for isolation. Finally, 0.1 mL aliquots from the selected dilutions were spread onto *MRS agar* plates to culture LAB colonies. Within 48 h after the inoculation, the plates were placed in an incubator, and the temperature was set at 37°C. After LAB growth on MRS agar, distinctive colonies were streaked onto new MRS agar plates using sterile inoculating needles. The streaks were then re-cultured at 37°C for a period of 1 to 2 days to purify the colonies (19).

### 2.3 Identification and characterization of lactic acid bacteria

The LAB was identified from buffalo milk fermented products through morphological and biochemical analysis. For the characterization of the LAB, freshly grown cultures were studied according to the previously reported methods (20, 21) with slight modifications. Gram staining, motility, catalase, oxidase, Triple Sugar Iron Agar, Indole, Methyl red, and Voges–Proskauer's Test were conducted.

#### 2.3.1 Gram staining

Gram staining was done using standard procedure, and Gram-positive bacilli/cocci and y shape were chosen for further characterization.

#### 2.3.2 Motility test

One colony of bacteria was inserted into the sulfide, indole, and motility media in a test tube. The test tube was incubated for 48 h at 37°C. The observation of the motility test is the growth of bacteria on the media. The bacteria that only grow around the inserted location show a negative result, while the bacteria that grow on the media surface or spread in the media show a positive result.

#### 2.3.3 Catalase test

The catalase test was conducted by adding a drop of a 3% solution of hydrogen peroxide to a glass slide on which a colony of bacteria was applied from a 24-h-old culture of each isolate (or directly on the Petri dish). NB: Catalase negative bacteria were subjected to further examination.

#### 2.3.4 Oxidase test

The test was conducted by the Filter Paper Spot Method. Use a loop to pick a well-isolated colony from a fresh bacterial plate and rub it onto a small piece of filter paper. Place 1 or 2 drops of 1% Kovács oxidase reagent on the organism smear. Observe for color changes.

#### 2.3.5 Voges–Proskauer's test

To the pre-sterilized glucose-phosphate broth tubes, test cultures were inoculated and incubated at 37°C for 48 h. After incubation, 10 drops of Barrit's reagent A were added and gently shaken, followed by the addition of Barrit's Reagent B. The development of pink color in the broth was taken as positive for the test.

#### 2.3.6 Triple Sugar Iron agar

Triple Sugar Iron (TSI) agar was inoculated with a pure culture of the isolates by stabbing through the center of the medium to the bottom of the tube and then streaked to the surface of the slant. Finally, the tube was incubated at 37°C for 24 h. LAB isolates ferment three sugar units, such as glucose, sucrose, and lactose, within the TSI medium and produces acids, which are indicated by color changes to yellow.

#### 2.3.7 Indole test

Inoculate the tube of tryptone broth with a small amount of a pure culture. Incubate at 35°C for 24 to 48 h. To test for indole production, add five drops of Kovács reagent directly to the tube. A positive indole test is indicated by the formation of a pink to red color (“cherry-red ring”) in the reagent layer on top of the medium within seconds of adding the reagent.

#### 2.3.8 Methyl red test

Sterilized glucose-phosphate broth tubes were inoculated with the test culture and incubated at 30°C for 48 h. After incubation, five drops of methyl red indicator were added to each tube and gently shaken. Red color production was taken as positive, and yellow color production was taken as negative for the test.

### 2.4 Molecular identification of microbes

#### 2.4.1 Extraction of total genomic DNA

Genomic DNA was extracted from 200 mg of bacterial biomass samples using the Qiagen AMP DNA Mini Extraction Kit following the manufacturer's instructions. The extraction process involved digestion of the sample with ATL Lysis Buffer and Proteinase K at 56°C for 1 h. Incubation with AL Buffer at 70°C for 10 min. DNA isolation using spin column centrifugation and subsequent washing with recommended buffers. Elution of purified DNA with TE buffer for downstream applications.

#### 2.4.2 PCR amplification

Polymerase Chain Reaction (PCR) amplification of the extracted DNA was performed using the PCR Dream Taq Green Master Mix (Thermo Scientific), with universal bacterial 16S rRNA primers 27F (5′-AGAGTTTGATCMTGGCTCAG-3′) and 1492R (5′-TACGGYTACCTTGTTACGACTT-3′). The reaction was set up according to the manufacturer's protocol, with the following conditions optimized for target amplification: Initial denaturation at 95°C, followed by 35 cycles of denaturation, annealing, and extension, with a final extension step at 72°C. Specific cycling parameters were adjusted according to the target DNA fragment.

#### 2.4.3 Agarose gel electrophoresis

Agarose gel electrophoresis was used to analyze the PCR products: A 1.5% agarose gel was prepared in 50X Tris-Acetate-EDTA (TAE) buffer. PCR products and the 100 bp DNA Ladder (Thermo Scientific) were loaded into the gel wells. Electrophoresis was conducted at 110 V for 30 min. DNA bands were visualized under UV light to confirm the size and integrity of the amplified products.

#### 2.4.4 PCR product verification

PCR amplification of the extracted DNA was performed using the PCR Dream Taq Green Master Mix (Thermo Scientific). The reaction was set up according to the manufacturer's protocol, with optimized conditions for target amplification.

#### 2.4.5 Gene sequencing

The purified PCR products were subjected to Sanger sequencing, which was performed by APICAL SCIENTIFIC SDN. BHD. Sequencing was carried out using standard protocols to confirm the identity and accuracy of the amplified DNA fragments.

### 2.5 Stress tolerance

The selected identified isolates from dairy products were further subjected to tests evaluating their tolerance to different pH levels, bile salt concentrations, and temperature ranges, following the probiotic activity assessment method reported by Menconi et al. (22).

#### 2.5.1 Tolerance to acidic pH values

Strains were grown in MRS broth at 37°C overnight, 0.1 mL aliquots of each active culture were adjusted to pH 5.0, 3.0, and 2.0 with 5 *N* HCl and incubated at 37°C for 3 h. Samples were taken every hour for 3 h, and the viable number of bacteria was enumerated by pour plate counts of all samples using 10-fold serial dilutions prepared in 0.1% peptone water. Simultaneously, bacterial growth was monitored by measuring absorbance with a spectrophotometer (Nova Spec II, Pharmacia) at 600 nm. All the experiments were replicated thrice.

#### 2.5.2 Bile tolerance

Strains were cultured overnight in MRS broth at 37°C. A saturated bile solution was prepared separately by dissolving powdered bile extract (Oxoid). The pH was maintained at 6.5 ± 0.2 using 1N HCl/NaOH during bile exposure. Bile solution was then sterilized by a 4-micron filter and was added to two of the cultures to achieve a final concentration of 0.3% and the second culture with 0% bile served as a control sample. The cultures were incubated at 37°C for 3 h and then every hour for 3 h. Viable counts of *Lactobacillus* strains were determined by pour plate counts of all the samples using 10-fold serial dilutions prepared in 0.1% peptone water. Simultaneously, bacterial growth was monitored by measuring absorbance with a spectrophotometer (Nova Spec II, Pharmacia) at 600 nm. All the experiments were replicated thrice.

#### 2.5.3 Resistance to temperature and sodium chloride

A basal MRS medium was utilized in these *in vitro* studies to cultivate the bacterial isolates. An overnight culture of each isolate served as the inoculum, with the cells being centrifuged and resuspended in 0.9% sterile saline. A 100-μL aliquot of the suspension was then inoculated into 10 mL of MRS broth in each test tube. Two incubation time points (2 h and 4 h) were evaluated for each of the variables: temperature and sodium chloride (NaCl) concentration. The temperatures tested were 5°C, 10°C, 15°C, and 45°C, and the NaCl concentrations tested were 2%, 4%, and 6.5% (w/v). The tubes were incubated with reciprocal shaking at the specified test temperatures and NaCl concentrations. At each time point, a sample from each tube was streaked onto MRS agar to assess the presence or absence of growth, which was used to confirm the viability of the strains. The turbidity of each tube was also recorded as an indicator of growth or no growth. Each treatment was tested in triplicate.

### 2.6 DPPH free radical scavenging ability

Free radical assays using 2,2-diphenyl-1-picrylhydrazyl hydrate (DPPH) (Sigma-Aldrich, St. Louis, MO, USA) was used for the evaluation of the radical-removing capabilities of LAB strains after vigorous mixing of 800 μL of a recently made 0.2 mM DPPH solution in 80% methanol with 400 μL of either intact cells or colony forming cells for 30 s (23–25). After this, it was kept at room temperature in the absence of light for 30 min, and the scavenging activity (%) was calculated from the absorbance at 517 nm (A517) relative to the methanol blank. Uninoculated MRS broth and PBS served as the control samples for the colony-forming cells and intact cell DPPH scavenging assays, and the percentage change in the scavenging activity was calculated using the following equation:


$$\begin{array}{l} DPPH\;scavenging\;capacity\;\left(\%\right)=\frac{\left(A\;control-A\;sample\right)}{\left(A\;control\right)}\times 100 \end{array}$$

### 2.7 Statistical analysis

The results of the statistical analysis were performed using Statistix 8.1 software. Data are reported as the mean ± standard deviation (SD). The data were subjected to a one-way ANOVA followed by LSD multiple comparison tests. Lowercase letters above the bars indicate a significant difference (*P* < 0.05).

## 3 Results

### 3.1 Isolation, morpho-physiological, and biochemical characterization of bacterial strains

A comprehensive isolation study identified 47 bacterial strains from traditional fermented dairy products, with 27 strains originating from yogurt samples and 20 strains from artisanal cheeses. Isolates were cultured on MRS agar plates to examine their morphological features. The findings of the motility test, Gram staining, and colony characteristics analysis are shown in Table 2. Staining revealed a positive character for all of the isolates, which produced a purple or violet color. The isolates obtained from MRS plates were rods, cocci, and v/y-shaped. Based on the observed shape, isolates were long, short, and bent rods, pink rods, cocci, tetra cocci, spherical, oval shapes, Y or V shapes, and also long filamentous forms presented in physiological test tables. Finding out if bacteria are motile or not and whether they have flagella that helps them move is the goal of the motility test. Motility testing of all isolates showed negative results except for Cm_1_ and Cm_6_. Colonies isolated from fermented dairy products were milky white, creamy, beige, yellowish, and light brown, as presented in Table 2.

**Table 2:** Results of the physiological test of yogurt and cheese isolates.

**Sample ID**	**Strains**	**Morphology**	**Motility**	**Colony appearance**
Y_1_	*Lactobacillus*	Rod shape	Non-motile	Milky white
	*Lactococcus*	Spherical shape	Non-motile	Creamy white
Y_2_	*Lactobacillus*	Rod shape	Non-motile	Milky white
	*Bifidobecterium*	Y shape	Non-motile	Milky white
	*Streptococcus*	Spherical shape	Non-motile	Creamy white
Y_3_	Mixture of *Lactobacillus*	Bent rods to short	Non-motile	Milky white
	*Streptococcus*	Spherical shape	Non-motile	Creamy white
Y_4_	*Lactobacillus*	Rod shape	Non-motile	Milky white
	*Actinomyces*	Long filamentous branches	Non-motile	Light brown
	*Lactococcus*	Spherical shape	Non-motile	White and Beige
Y_5_	*Bifidobecterium*	Y and v shape	Non-motile	Cream to white
	*Lactobacillus*	Rod shape	Non-motile	Milky white
Y_6_	*Lactobacillus*	Rod shape	Non-motile	Milky white
	*Lactococcus*	Cocci/spherical	Non-motile	Creamy gray
	*Streptococcus*	Spherical shape	Non-motile	Creamy white
Y_7_	*Bifidobecterium*	Y shape	Non-motile	Creamy white
	*Lactobacillus*	Rod shape	Non-motile	Milky white
	*Actinomyces*	Long filamentous	Non-motile	Beige white
Y_8_	*Lactobacillus*	Rod shape	Non-motile	Milky white
	*Lactococcus*	Spherical or ovoid shape	Non-motile	Creamy white
	*Lactococcus*	Cocci	Non-motile	Creamy white
Y_9_	*Lactobacillus* mix strain	Rod shape (long & short rods)	Non-motile	Milky white
	*Bifidobecterium*	Y shape	Non-motile	Creamy white
	*Lactococcus*	Spherical or ovoid shaped	Non-motile	creamy yellow
Y_10_	*Lactobacillus*	Rod shape	Non-motile	Milky white
	*Streptococcus*	Spherical	Non-motile	Creamy white
Cm_1_	*Lactococcus*	Mono and streptococci	Non-motile	Creamy white
	*Bacillus*	Long rod	Motile	White
Cm_2_	*Lactobacillus*	Long and short Rod	Non-motile	Milky white
	*Bifidobecteria*	Y shape	Non-motile	Beige and white
Cm_3_	*Lactococcus*	Monococci	Non-motile	Creamy white
	*Lactobacillus*	Short rod	Non-motile	Milky white
Cm_4_	*Bacillus*	Long Rod	Motile	Creamy gray
	*Streptococcus*	Spherical shape	Non-motile	Creamy white
Cm_5_	*Lactococcus*	Round shape/small cocci	Non-motile	Yellowish
	*Lactobacillus*	Rod shape	Non-motile	White
Cc_1_	*Bifidobecterium*	Y or v shape	Non-motile	Cream to white
	*Lactococcus*	Long chain Cocci/spherical	Non-motile	Creamy
Cc_2_	*Lactococcus*	Tetracocci	Non-motile	Creamy white
	*Lactobacillus*	Rod shape	Non-motile	Milky white
Cc_3_	*Lactobacillus*	Rod shape	Non-motile	White
	*Lactococcus*	Spherical or ovoid shape	Non-motile	Gray white
Cc_4_	*Lactobacillus*	Light pink rods	Non-motile	Beige
	*Lactococcus*	Spherical or ovoid shaped	Non-motile	Creamy
Cc_5_	*Lactobacillus*	Rod shape	Non-motile	Milky white
	*Lactococcus*	Diplococci	Non-motile	Creamy white

### 3.2 Biochemical characterization of LAB isolates

To identify the specific strain of LAB, a series of biochemical tests was conducted, including Gram staining, the assessment of catalase and oxidase, methyl red, triple sugar iron, gas generation, and fermentation. These are the most common tests used to identify lactic acid bacteria. Surprisingly, no single strain of yogurt and cheese samples was found to be positive for the catalase and oxidase, as presented in Tables 3, 4. The VP test checks the production of acetyl methyl carbinol by any microbe through glucose fermentation. Most of the isolates showed negative results on the VP test. At the same time, some isolated strains of some samples, such as Y_4_, Y_6_, and Cc_1_, showed positive results on the VP test in Tables 3, 4. Except for one strain, every single isolate fermented glucose, lactose, and sucrose, turning yellow upon the slant/butt, and every single strain tested negative for hydrogen sulfide formation of Y_4_, and Y_7_ showed K/A (alkaline over acid) on TSI that means only glucose was metabolized (Tables 3, 4). Glucose fermentation to pyruvic acid and subsequent oxidation to other acids (e.g., lactic, acetic, and formic acids) leads to a reduction in pH; this is the basis of the methyl red test. The subsequent reddening of the methyl red indicator shows a favorable response. Some isolated strain from samples Y_1_, Y_2_, Y_3_, Y_4_, Y_6_, Y_7_, Y_9_, Y_10_, Cm_1_, Cm_3_, Cm _4_, Cc_1_, Cc _2_, Cc _4_, and Cc_5_ tested positive for methyl red test. The remaining isolates tested negative for the methyl red test presented in Tables 3, 4. The isolates under study did not possess the characteristic of producing indole from tryptophan by “tryptophanase.” Therefore, the addition of Kovac's reagent did not lead to the formation of a deep red color on the surface layer; it remained yellow as it was initially. It means that all isolated strains showed negative results on the indole test (Tables 3, 4).

**Table 3:** Results of biochemical tests of yogurt isolates.

**S#**	**Isolated strains**	**Gram staining**	**Catalase test**	**Oxidase test**	**VP test**	**Indole test**	**TSI test**	**Methyl red test**
Y_1_	*Lactobacillus*	(+)	-	-	-	-	A/A	-
	*Streptococcus*	+	-	-	-	-	A/A	+
Y_2_	*Lactobacillus*	+	-	-	-	-	A/A	-
	*Bifidobecterium*	+	-	-	-	-	A/A	+
	*Streptococcus*	+	-	-	-	-	A/A	+
Y_3_	Mix strains of *Lactobacillus*	+	-	-	-	-	A/A	-
	*Streptococcus*	+	-	-	-	-	A/A	+
Y_4_	*Lactobacillus*	+	-	-	-	-	A/A	-
	*Actinomyces*	+	-	-	-	-	K/A	+
Y_5_	*Bifidobecterium*	+	-	-	-	-	A/A	+
	*Lactobacillus*	+	-	-	-	-	A/A	-
Y_6_	*Lactobacillus*	+	-	-	-	-	A/A	-
	*Lactococcus*	+	-	-	-	-	A/A	+
	*Streptococcus*	+	-	-	-	-	A/A	+
Y_7_	*Bifidobecterium*	+	-	-	-	-	A/A	+
	*Lactobacillus*	+	-	-	-	-	A/A	-
	*Actinomyces*	+	-	-	-	-	K/A	+
Y_8_	*Lactobacillus*	+	-	-	-	-	A/A	-
	*Lactococcus*	+	-	-	-	-	A/A	-
	*Streptococcus*	+	-	-	-	-	A/A	+
Y_9_	*Lactobacillus* mix strain	+	-	-	-	-	A/A	-
	*Bifidobecterium*	+	-	-	-	-	A/A	+
	*Lactococcus*	+	-	-	-	-	A/A	+
Y_10_	*Lactobacillus*	+	-	-	-	-	A/A	-
	*Streptococcus*	+	-	-	-	-	A/A	+

+, Positive; -, Negative.

**Table 4:** Results of the biochemical test of cheese isolates.

**S#**	**Isolated strains**	**Gram staining**	**Catalase test**	**Oxidase test**	**VP test**	**Indole test**	**TSI test**	**Methyl red test**
Cm_1_	*Lactococcus*	+	-	-	-	-	A/A	-
	*Bacillus*	+	-	-	-	-	A/A	+
Cm_2_	*Lactococcus*	+	-	-	-	-	A/A	-
	*Bifidobecteria*	+	-	-	-	-	A/A	+
Cm_3_	*Lactococcus*	+	-	-	-	-	A/A	+
	*Lactobacillus*	+	-	-	-	-	A/A	+
Cm_4_	*Lactobacillus*	+	-	-	-	-	A/A	-
	*Streptococcus*	+	-	-	-	-	A/A	+
Cm_5_	*Lactococcus*	+	-	-	-	-	A/A	-
	*Lactobacillus*	+	-	-	-	-	A/A	-
Cc_1_	*Bifidobacterium*	+	-	-	-	-	A/A	+
	*Lactococcus*	+	-	-	+	-	A/A	+
Cc_2_	*Lactococcus*	+	-	-	-	-	A/A	-
	*Lactobacillus*	+	-	-	-	-	A/A	+
Cc_3_	*Lactococcus*	+	-	-	-	-	A/A	-
	*Lactobacillus*	+	-	-	-	-	A/A	+
Cc_4_	*Lactobacillus*	+	-	-	-	-	A/A	+
	*Lactococcus*	+	-	-	-	-	A/A	+
Cc_5_	*Lactobacillus*	+	-	-	-	-	A/A	-
	*Streptococcus*	+	-	-	-	-	A/A	+

+, Positive; -, Negative.

### 3.3 Species identification

**Figure 1:**
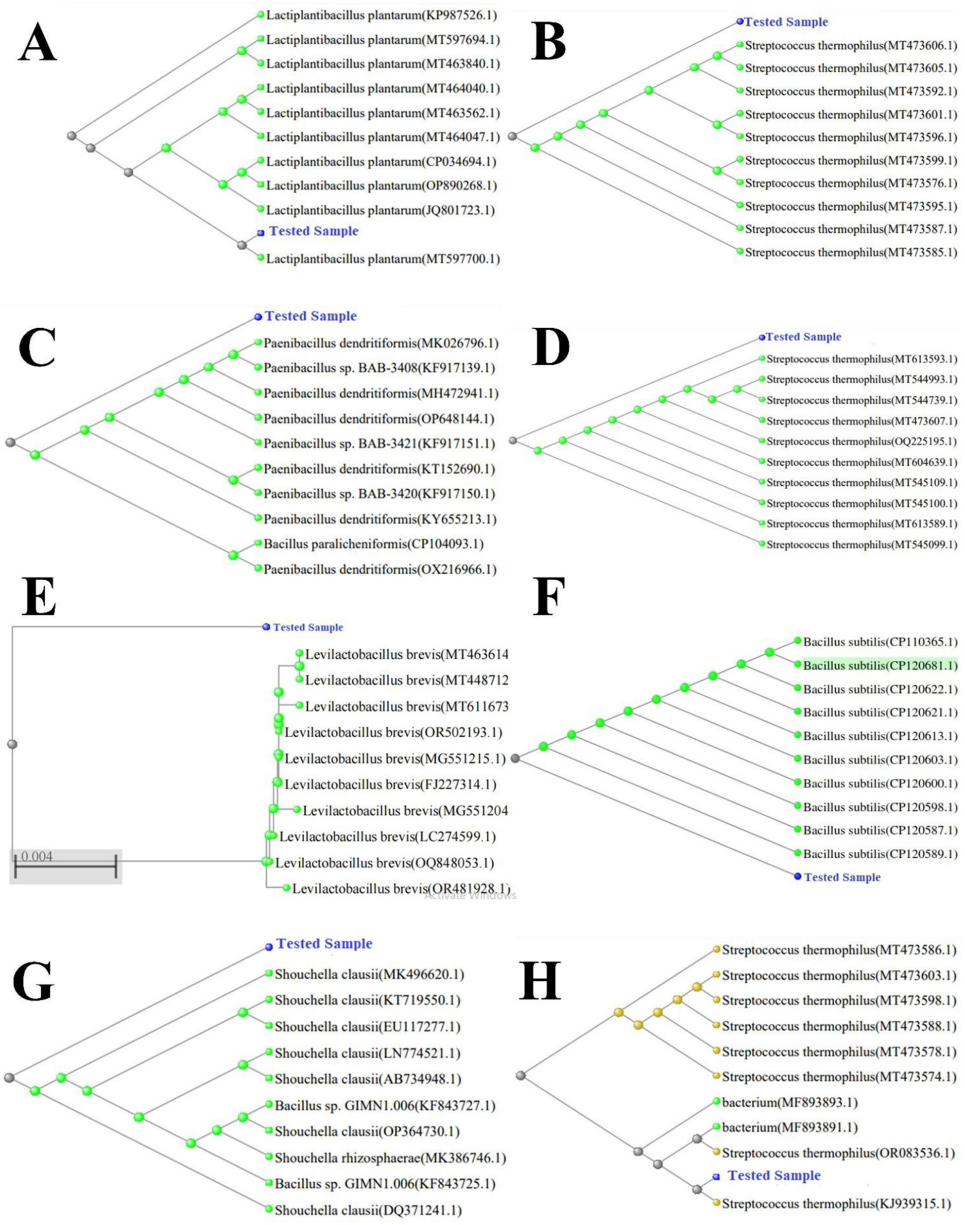
Phylogenetic tree with bootstrap values. Tree was constructed using Neighbor-Joining (NJ) method. **(A)** *L. plantarum* (accession PP565108), **(B)** *S. thermophilus* (accession PP565114), **(C)** *B. dendritiformis* (accession PP565143), **(D)** *S. thermophilus* (accession PP565141), **(E)** *L. brevis* (accession PP549910), **(F)** *B. subtilis* (accession PP89025), **(G)** *S. clausii* (accession PP565148), and **(H)** *S. thermophilus* (accession PP565129).

The characteristics of the bacterial isolates from the study were examined for their biochemical identification of species. Out of these, eight strains underwent species identification through sequencing in the Gene-Bank database. A phylogenetic tree was developed after the identification (Figure 1), which revealed the connections of isolate Y_1_ to the genus *Lactobacillus* with 98.7% similarity to *L. plantarum* MT597700.1 strain. Strain Y_6_ had associations with the genus *Lactococcus*, with 98.9% similarity to the *S. thermophilous* MT473585.1 strain. Y_9_ had a similarity to genus *Bacillus* and showed 94.9% similarity to the *B. dedritiformis* OX216966.1 strain, while Cc_1_ had connections with the *S. thermophilus* strain MT544739.1. Cm_1_ was revealed to be related to the genus *Bacillus* with 98.9% similarity to *B. subtilis* CP120681.1 strain. Cc_3_ isolated bacterial strain was matched with the lactobacillus strain *L. brevis* OQ848053.1. The complete phylogenetic tree of all the studied strains is shown in Figure 1.

### 3.4 Bile salt stress tolerance potential of isolates

**Figure 2:**
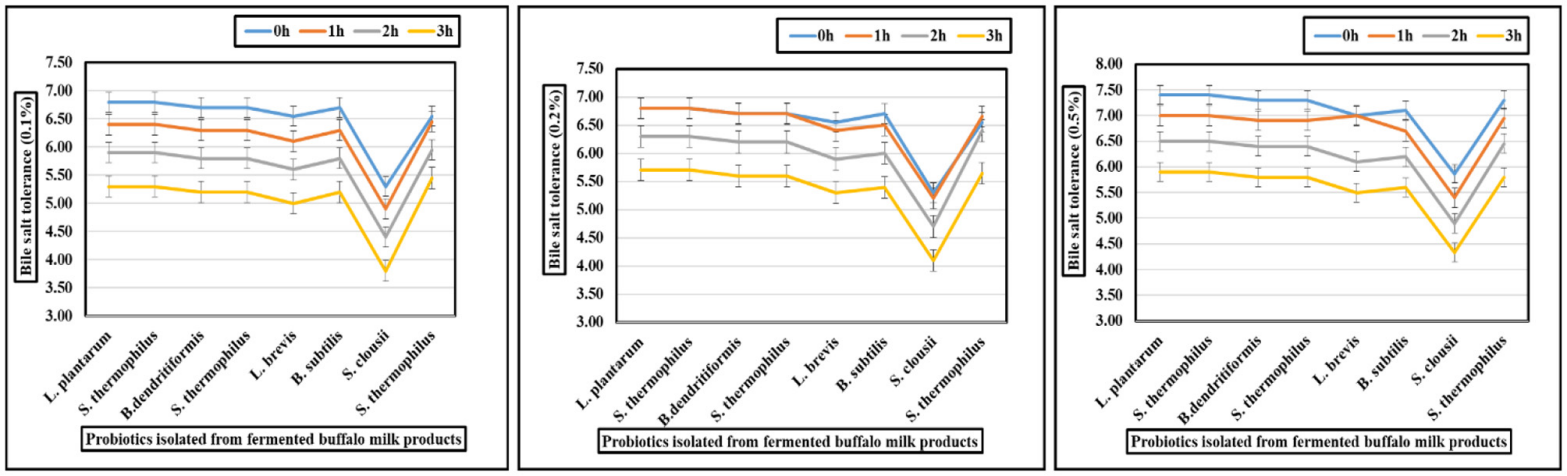
Graphs showing bile (0.1, 0.2, and 0.5%) tolerance of probiotic isolated from fermented buffalo milk products.

**Figure 3:**
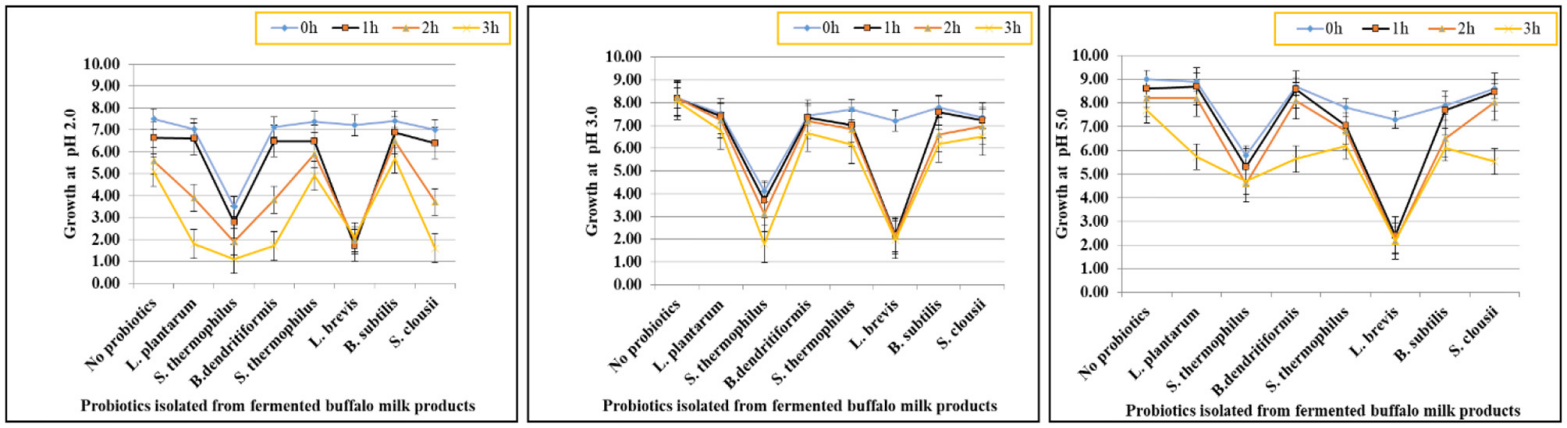
Graphs showing tolerance of probiotic isolated strains at pH 2, 3, and 5.

**Figure 4:**
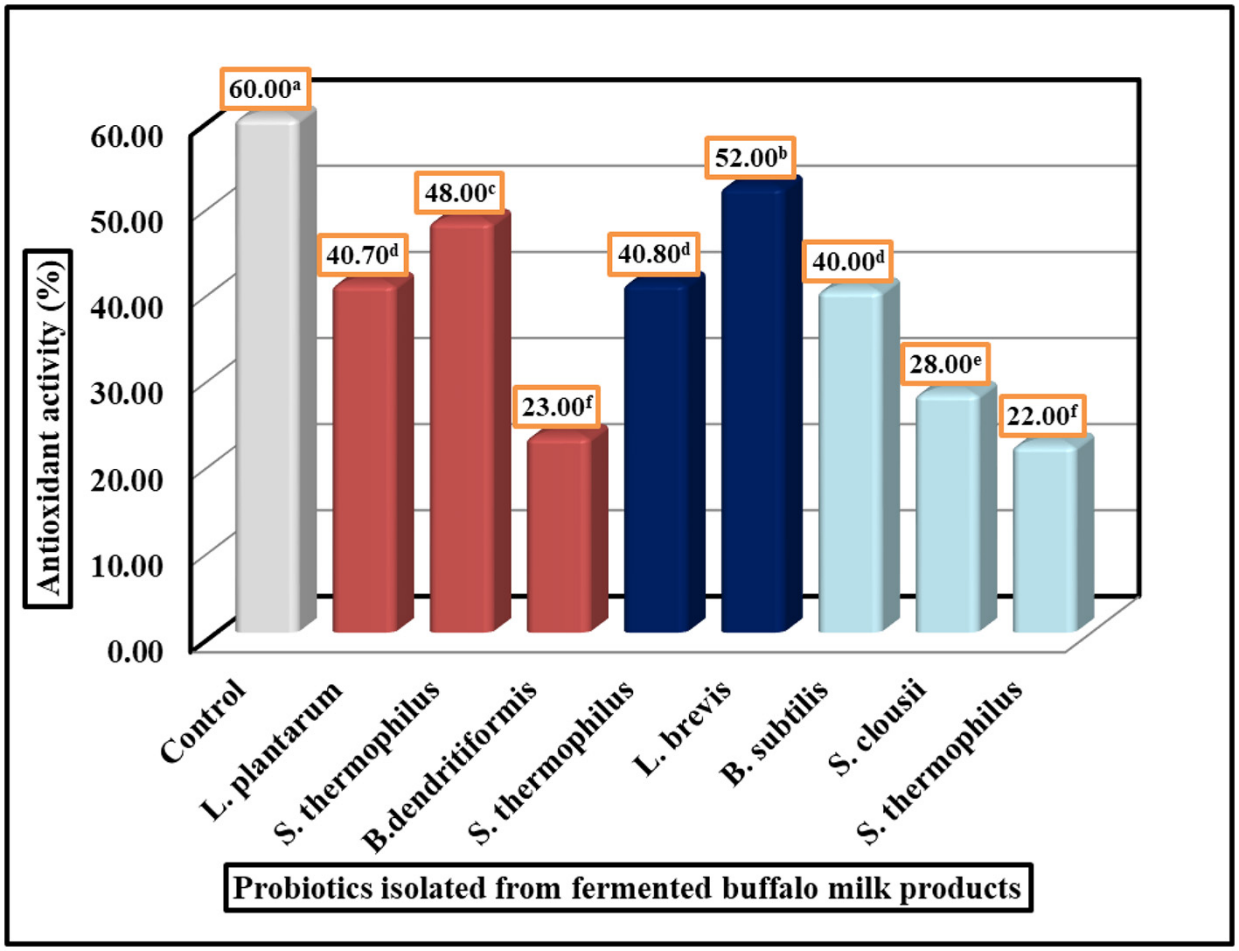
Antioxidant activity of probiotic isolated strains by DPPH assay. LSD All-Pairwise Comparisons Test (0.05) = 05, SE ± 0.80.

The gastrointestinal stress tolerance and functional antioxidant capacity of isolated probiotic strains were systematically evaluated through a series of *in vitro* assays simulating physiological challenges. As shown in Figures 2–4 and Table 4, the isolates exhibited significant strain-dependent variations in their ability to withstand bile salts, acidic pH, temperature fluctuations, osmotic stress, and their free radical scavenging potential. The bile tolerance results demonstrated a concentration and time-dependent decline in the viability of all tested bacterial strains (Figure 2). At 0.1% bile concentration, bacterial survival remained relatively high after 3 h of exposure. *L. plantarum* exhibited the highest bile resistance, maintaining optical densities corresponding to viable counts between 6.4 and 6.5 log10 CFU/mL, while *S. clausii* showed the lowest tolerance, with final counts ranging from 3.7 to 3.9 log10 CFU/mL. At 0.3% bile, a moderate but noticeable reduction in viability was observed across all strains. *L. plantarum* continued to display superior tolerance, retaining viable counts of 5.7–5.8 log10 CFU/mL, followed by *B. subtilis* at *5.4–5.5* log10 CFU/mL. In contrast, *S. clausii* exhibited a further decline in viability, with counts ranging between 4.0 and 4.2 log10 CFU/mL. At the highest concentration, 0.5% bile, the inter-strain variability became most evident. *L. plantarum* maintained strong resistance, sustaining viable counts between 5.9 and 6.0 log10 CFU/mL even after 3 h. Conversely, *S. clausii* experienced a significant drop in viability, decreasing to 4.2 to 4.4 log10 CFU/mL, which reflects a reduction of approximately 2.2 log units from its initial value.

### 3.5 Low pH stress tolerance potential of isolates

The acid tolerance of eight probiotic strains was assessed at pH 2.0, 3.0, and 5.0 over a 3-h period. *L. plantarum, S. thermophilus* (Strain 1), and *B. dendritiformis* showed the highest acid tolerance, retaining viability above 5.0 log10 CFU/mL at pH 2.0 after 3 h, with survival rates between 66.8% and 69.2%. At pH 5.0, all three exceeded **7.6** log10 CFU/mL, maintaining survival rates above 83%. *L. brevis* and *S. thermophilus* (Strain 2) showed moderate tolerance, dropping to 1.6–1.9 log10 CFU/mL at pH 2.0 (survival: 22–27%), but remained viable at pH 5.0 (final counts: 5.3–5.9 log10 CFU/mL). *B. subtilis, S. clausii*, and *S. thermophilus* (Strain 3) exhibited low acid tolerance, with survival below 2.2 log10 CFU/mL at pH 2.0 and reduced growth even at pH 5.0 (final counts: 2.1–4.7 log10 CFU/mL). Survival rates were as low as 21–31% at pH 2.0. Statistical analysis (one-way ANOVA, *p* < 0.05) confirmed significant differences in acid survival across strains and pH levels. *L. plantarum, S. thermophilus* (Strain 1), and *B. dendritiformis* were identified as the most acid-resistant strains. These findings are presented visually in Figure 3, which illustrates the differential survival patterns across pH conditions and time points.

### 3.6 Temperature and salt tolerance potential of isolates

The tested isolates Y_1_, Y_9_, Cc_3_, and Cm_4_ showed growth at temperature range 5, 10, 15, and 45°C, while Y_6_, Cc_1_, isolated strain *S. thermophilus*, and Cm_1_ isolated strains *B. subtilis* could grow at 10 to 45°C but showed partial growth at 5°C. The ability to thrive in high conditions is a good characteristic of lactic acid production, which decreases the chances of contamination by other microorganisms (Table 5). The ability to grow in different salt conditions is also an *in vitro* test guideline to evaluate when selecting potential probiotic bacteria. The selected identified strains were determined for salt tolerance. All isolates can grow in a 2, 4, and 6.5% salt range. The results indicated positive growth rates for all six isolated strains mentioned in Table 5. The isolated strains could resist a 2–6.5% w/v concentration of NaCl in MRS broth.

**Table 5:** Temperature and salt stress tolerance potential of isolates.

**Isolate code**	**Identified strain**	**Growth at temperature (°C)**				**Growth at salt (%w/v)**		

		**5**	**10**	**15**	**45**	**2%**	**4%**	**6.5%**
Y_1_	*L. Plantarum*	+	+	+	+	+	+	+
Y_6_	*S. thermophilus*	-	+	+	+	+	+	+
Y_9_	*B. dendritiformis*	+	+	+	+	+	+	+
Cc_1_	*S. thermophilus*	-	+	+	+	+	+	+
Cc_3_	*L. brevis*	+	+	+	+	+	+	+
Cm_1_	*B. subtilis*	-/+	+	_+_	+	+	+	+
Cm_4_	*S. clausii*	+	+	+	+	+	+	+
Cm_5_	*S. thermophilus*	-/+	+	+	+	+	+	+

(+), Growth present; (-), Growth absent.

### 3.7 DPPH free radical scavenging ability of selected isolates

The antioxidant activity of probiotic strains was evaluated using the DPPH radical scavenging assay. The results demonstrate significant variation in antioxidant potential among the tested strains, with values ranging from 22% to 52% inhibition (Figure 4). Strain 5 (*L. brevis*) exhibited the highest antioxidant activity at 52%, followed by Strain 2 (*S. thermophilus*) at 48% and Strain 4 (*S. thermophilus*) at 40.80%. In contrast, Strain 3 (*B. dendritiformis*) showed the lowest activity at 22%, with Strain 6 (*B. subtilis*) and Strain 8 (*S. clausii*) also displaying relatively weak antioxidant capacity at 23% and 28%, respectively. The remaining strains demonstrated intermediate levels of antioxidant activity. Statistical analysis confirmed the reliability of these measurements, with a standard error of ± 0.80 and an LSD All-Pairwise Comparisons Test indicating meaningful differences between most strains. These findings reveal that certain probiotic isolates, particularly *Lactobacillus brevis* and specific *S. thermophilus* strains, possess substantial antioxidant properties that may be valuable for developing functional foods or dietary supplements aimed at oxidative stress reduction. The results provide a basis for selecting optimal strains for further investigation and potential commercial applications where antioxidant activity is desired.

## 4 Discussion

### 4.1 Morphological and physiological characteristics of the isolates

The LAB strains were isolated from the collected samples of yogurt and other fermented dairy products. Isolated from the materials, 47 strains were further examined for microscopic, physiological, and biochemical traits before their molecular characterization. A total of 20 bacterial strains (rod-shaped, cocci, and long filaments) were identified, with the majority of the bacterial colonies being pure white. This is due to the fact that color is contingent upon the isolate's origin (26–28). Furthermore, it has also been reported that the morphology of LAB colonies isolated from buffalo milk is oval to round-shaped with a yellow zone around the colony (29, 30). In this study we found four shapes of the bacteria and the first group comprises 20 isolates, the second group comprises 19 isolates in the form of cocci, diplococci, and *streptococcus*, third group consisted of six strains in the shape of “v” and “y-shaped” bacteria, and the fourth group contained only one bacterial strain with long filamentous shape (Tables 2, 3). Many studies, including (31), have characterized LAB isolates from traditional fermented mare milk, and our results align with these prior reports. A study by (28) reported that LAB collected from yogurt were Gram-positive. Moreover, another study (32) also found that LAB from buffalo milk curd belonged to *Lactobacillus* sp. and *Streptococcus* spp. Similarly, the study by (33) reported similar results, indicating that approximately 68% of the LAB isolates from fresh and fermented milk were Gram-positive. It has also been reported previously that 84 out of the 96 LAB from bovine milk were found as Gram-positive, with shapes ranging from round to rod-shaped (34, 35). Thakur et al. also reported similar results after analyzing samples of dairy and non-dairy products (36). This finding implies that flagella are absent from all LAB isolates. The lactic acid bacteria isolates from bovine milk curd are non-motile (28). The genus *Lactobacillus* is characterized by non-motile lactic acid bacteria (37).

### 4.2 Biochemical characterization of LAB isolates

#### 4.2.1 Catalase and oxidase test

Lactic acid bacterial cells isolated from fermented dairy products have been the subject of several reports about their distinctive catalase test. All of the isolates tested negative for catalase and oxidase in our study. It has been found that specific Lactobacillus isolates, including *Lactobacillus delbrueckii* ssp. *Bulgaricus*, isolated from yogurt and other medicines and probiotics also exhibited no oxidase and catalase production (36), with only negative reaction observed in *Lactobacillus* isolates during experiments (38, 39). Buffalo milk microbial diversity was also investigated. Isolates were Gram-positive, oxidase-negative, catalase-negative, and characterized by tiny, localized colonies, all of which are hallmarks of LAB (40). Further identification of *Lactobacilli* species in local raw milk revealed that the catalase test was negative for all species (41). Similar catalase oxidase-negative reactions have been reported previously; they also reported that LAB isolates from dadih were non-motile, catalase, and oxidase-negative (42, 43). In addition, the study by (44) reported that the lactic acid bacteria in dadih exhibit negative catalase and oxidase activity. A further investigation found that 52 out of 70 lactic acid bacterial isolates in bovine milk from the North Sumatera River did not contain catalase (29). Additionally, it has also been previously reported that 99% of the LAB isolated from the milk of buffalo were catalase-negative (29, 45).

#### 4.2.2 Voges–Proskauer's test

The presence or absence of acetyl methyl carbinol during glucose fermentation is shown by the VP test. The VP test came out negative for the majority of the isolates. These results agreed with those found by (46), who reported that *L. plantarum* from kimchi-fermented products exhibited an adverse reaction. *Lactobacillus* isolates from yogurt exhibited an adverse reaction, as reported by (47). In the Voges Proskauer test reaction conducted by (48), the prevalent characteristics of *Lactobacillus* species yielded negative results. The current investigation results are comparable to those of (49), who reported that the Voges–Proskauer test did not induce any color changes in LAB strains. Similarly, Ngene et al. (50) noted that the Voges–Proskauer's test yielded negative results for the *L. lactis, L. brevis*, L. *fermentum, L. casei*, and *L. plantarum* strains in yogurt. The single isolated isolates, including Y_4_, Y_6_, Y_7_, and Cc_1_, exhibited positive results on the VP test in our research. An additional study indicated that certain LAB isolates exhibited positive and negative reactions (51).

#### 4.2.3 TSI test

The outcomes of this investigation on TSI were favorable. Fermented milk carbohydrate fermentation patterns have been used by certain studies to identify Lactobacillus species. Findings were the same when analyzed by (52), who characterized LABs and *bifidobacterium* from yogurt. Other studies, including (53), also reported comparable TSI results for LAB isolates, such as *Lactococcus lactis* subsp. lactis, *L. acidophilus, L. plantarum, L. fermentum*, and *L. lactis*. Manolopoulou et al. (54) found 20 distinct strains of *Lactobacillus delbrueckii* ssp. *bulgaricus* in the fermented fructose, glucose, galactose, lactose, and sucrose extracts from typical Feta cheese (54). Certain researchers have identified *Lactobacillus* species in fermented milk through carbohydrate fermentation patterns. Six different species of lactic acid bacteria have been isolated from dadih, an Indonesian food made from buffalo milk: *L. lactis, L. mesenteroides, L. plantarum, L. casei, and L. paracasei* (55). Similarly, six different species of lactic acid bacteria, *L. bulgaricus, L. plantarum, L. lactis, L. acidophilus, L. brevis*, and *L. rhamnosus*, were isolated from buffalo milk in India. Carbohydrate fermentation patterns were utilized in both cases to identify Lactobacillus species in fermented milk (56, 57).

#### 4.2.4 Methyl red and indole test

Similar results to our study regarding positive MR tests were also previously reported by (58), who found that LAB in the fermented products showed a positive reaction. The methyl red test was negative for those isolates (59). Traditional fermented foods, including dosa butter, appam batter, buttermilk, yogurt, and cabbage, were found to contain *Lactobacillus* bacteria that showed a negative response (60). These current results are in line with the reported work of (61), who observed that *L. acidophilus* from the yogurt samples showed a negative reaction. It was determined that certain LAB isolates from fermented foods exhibited positive reactions to the methyl red test, while others exhibited negative reactions (1). According to (62), adverse reactions were exhibited by *L. bulgaricus* and *L. fermentum* from curd and milk. An additional study revealed that *L. lactis, L. brevis, L. fermentum, L. casei*, and *L. plantarum* from yogurt exhibited a negative reaction. The isolates under investigation did not exhibit the ability to synthesize indole (50). The same results were documented by (63). Additionally, LAB isolated from regional cow milk kefir and found to be indole-negative have been described (64).

### 4.3 Phylogenic tree

Recent studies have used 16S rDNA phylogenetic analysis to isolate lactic acid bacteria (LAB) with probiotic potential from fermented foods (1, 65). Our 16S rRNA gene analysis similarly identified *Lactobacillus* strains phylogenetically related to *L. fermentum, L. acidophilus, L. casei*, and *L. buchneri*. Other studies have likewise characterized *Lactobacillus spp*. with probiotic traits via 16S rRNA sequencing (58, 66–70). Coulibaly et al. (70) further applied 16S rDNA sequencing to compare 12 LAB strains, including *Pediococcus* (*P. acidilactici* and *P. pentosaceus*) and *Lactobacillus*. These strains were selected based on probiotic functionality, stability, and safety criteria (70). The Bacillaceae family (*Firmicutes*) comprises mesophilic, Gram-positive bacteria (71). Notably, *B. dendritiformis* strains exhibit gastrointestinal adaptability, a key probiotic trait. In this study, we successfully isolated *B. dendritiformis* as a candidate probiotic.

### 4.4 Stress tolerance potential

#### 4.4.1 Bile salt and acid tolerance

This study evaluated the bile salt and acid tolerance of eight probiotic strains isolated from fermented buffalo milk products, including *Lactobacillus plantarum, Streptococcus thermophilus, Bacillus dendritiformis, Lactobacillus brevis, Bacillus subtilis*, and *Souchella clausii*. *B. subtilis* and *B. dendritiformis* showed the highest bile salt tolerance (5.4–6.0 log10 CFU/mL at 0.5% bile), while *L. plantarum* demonstrated superior resistance (5.9–6.5 log10 CFU/mL) and *S. clausii* showed moderate survival (4.2–4.4 log10 CFU/mL). *S. thermophilus* exhibited the lowest bile tolerance due to its lack of bile salt hydrolase activity (72). In terms of acid tolerance, *L. plantarum, S. thermophilus* (Strain 1), and *B. dendritiformis* displayed the greatest acid resilience (>5.0 log10 CFU/mL at pH 2.0), while *B. subtilis* and *S. clausii* showed low tolerance (< 2.2 log10 CFU/mL), supported by bile salt hydrolase production and spore formation (37). *L. brevis* and *S. thermophilus* showed moderate acid tolerance, while *B. dendritiformis* and *S. thermophilus* exhibited the lowest acid resistance, particularly at pH 2.0 (73). Overall, *L. plantarum, B. dendritiformis*, and *S. thermophilus* (Strain 1) were most promising for gastrointestinal survival, showing dual bile/acid tolerance.

#### 4.4.2 Temperature and NaCl tolerance

For LAB fermentation to work properly, temperature is also crucial. Yogurt is best fermented at 42°C and stored at 4°C, so it is important that the probiotic strain you choose can adjust to these temperatures (74). As shown in Table 4, the isolated strains showed positive growth at 10–45 °C, but only *S. thermophilus* (PP565141) showed negative growth at 50°C. The detected strains were able to adjust effectively to the temperature of fermented foods and dairy, as the growth was still excellent. In (75), a total of 14 samples of fermented milk and products were studied, and it was observed that pH values were approximately 4.0. Since temperature is a critical prerequisite for bacterial development, the fact that LAB were able to live in the specified temperature range (25–40°C) suggests that they may be able to survive in the human gut temperature. The only exception to this is *L. cremoris*, whose growth was halted at 0°C. To mimic the typical temperature, range of the human body, a certain range was determined. Since the growth/viability of probiotics during storage and usage is a crucial component in determining their functioning, this element is very significant in evaluating the efficacy of probiotics (76). In addition, the fact that all of the isolates were able to withstand concentrations of NaCl ranging from 4% to 6% further suggests that they may be able to withstand the severe circumstances and bile salt found in the colon (77). The strain's survival under harsh conditions suggests strong potential for probiotic applications. According to (78), not only LAB in fermented food, but some species of Bacillus also have potential probiotics. Despite *B. clausii*'s long history of usage, the literature more effectively promotes the therapeutic characteristics of other probiotics, such as *Lactobacillus* sp. and *Bifidobacterium* sp. (79–81). Among the four selected strains, all of them could survive well at 0.8% bile salts, three of them could tolerate up to 1.8% bile salts (82).

### 4.5 Scavenging of DPPH radicals

Because of its ease of use, rapidity, sensitivity, and reproducibility, the DPPH radical scavenging technique is widely employed to evaluate antioxidant activity (23–25, 83). The basic idea behind the test is to convert an ethanolic DPPH solution to the non-radical form DPPH-H in the presence of an antioxidant that donates hydrogen. Diphenyl picryl hydrazine, a stable radical, may be transformed from purple to yellow by the antioxidant. The following figure serves as an illustration of this. The tested isolates (*Streptococcus spp*., *Bacillus spp*., and *Lactobacillus spp*.) showed DPPH scavenging ranging from 22% to 52% (Figure 4), with *L. brevis* Cc_3_ exhibiting the highest activity (52%), which agrees with the value given by (84). Similarly, the study by (85) recorded DPPH levels of ~50% for 11 different strains of *Lactobacillus*. At 8 log CFU/mL, *B. subtilis* P223 showed 41.52% antioxidant activity in the DPPH experiment, while *B. subtilis* ATCC 6633 showed 22.45%. *B. subtilis* ${\text{Y}}_{9^{\text{′}}}$s DPPH scavenging (22.45%) aligned with *B. subtilis* ATCC 6633 (86), suggesting conserved antioxidant mechanisms among *Bacillus* strains. Our research showed that *L. plantarum* has a 40.70% antioxidant activity. Ten strains of *L. plantarum* were previously tested for resistance to hydrogen peroxide and hydroxyl and 2,2-diphenyl-1-picrylhydrazyl (DPPH) free radicals *in vitro*. The strains were selected from traditional fermented foods in China. At 10 CFU/ml, *L. plantarum* C88 showed the strongest DPPH and hydroxyl radical scavenging capabilities, with inhibition rates of 53.15% and 44.31%, respectively. *L. plantarum* Y_1_'s activity (40.70%) resembled that of *L. plantarum* C88 (53.15%) from fermented dairy (87), highlighting cross-regional consistency in *Lactobacillus* antioxidants. The free radical 2,2-diphenyl-1-picrylhydrazyl (DPPH) was scavenged by the isolates in the *in vitro* antioxidant tests. *L. plantarum* IH16L had the greatest hydroxyl radical scavenging activity (82.25 ± 1.60%), compared to the other isolates, whereas *L. plantarum* IH26L had the lowest (35.60 ± 4.50%). Between 7.22 ± 0.04% and 21.63 ± 1.32%, the microorganisms exhibited superoxide radical scavenging capabilities. These findings suggest a correlation between DPPH and hydroxyl radical scavenging, as seen in *L. plantarum* IH16L (82.25% hydroxyl scavenging) (88). The traditional yogurt starter is *S. thermophilus*. When tested *in vitro*, ST-EPS2 outperformed the other compounds in terms of antioxidant activity. Enhanced free radical scavenging activities (ABTS, DPPH, and ${\text{O}}_{2}^{-}$) were seen in the barley fluid fermented with *S. thermophilus* 7G10, which aligns with our results (89). Nevertheless, the juice or products' antioxidant activity can be enhanced by altering the form and constituents of phenolic compounds by LAB fermentation (90). Clinical evidence establishes *S. clausii* as an effective antidiarrheal probiotic across age groups. Our isolate *S. clausii* Cm_4_ demonstrated significant antioxidant capacity (28% DPPH scavenging), comparable to literature reports for strain CSI08, which shows immunomodulation via U937 macrophage activation and cyto-protection in *C. elegans* oxidative stress models. These findings align with our observations, reinforcing its dual role in antioxidative and immunomodulatory functions. These documented strain-specific properties validate *S. clausii* as a therapeutically promising probiotic with dual antioxidant-immunomodulatory activity (11). A total of 27 studies found evidence of *B. dendritiformis*'s possible effects. A study by (91) demonstrated that the levels of glutathione s-transferase (GST) and glutathione reductase (GR) were enhanced when *B. subtilis* was used in conjunction with fish. An additional investigation demonstrated that dietary supplementation with *B. dedritiformis* Dahb1 could enhance the innate immune system, which reduces the oxidative stress associated with the deposition of ammonia in tissues and blood (92).

## 5 Conclusion

This study highlights Pakistani fermented buffalo milk as a rich source of diverse probiotics, including both lactic acid bacteria (*Lactobacillus* and *Streptococcus*) and hardy non-LAB strains (*Bacillus* spp.). Among the notable discoveries, *L. plantarum* Y_1_ demonstrates remarkable survival in harsh gastrointestinal conditions, with a strong tolerance to pH 2.0 and 0.5% bile, indicating a high potential for gut colonization. Another standout strain, *L. brevis* Cc_3_, exhibits a rare combination of bile resistance and significant antioxidant activity, scavenging 52% of DPPH radicals. All tested strains display adaptability to industrial conditions, thriving across a wide temperature range (4–45°C) and tolerating NaCl, though *S. thermophilus* ${\text{Cc}}_{1^{\text{′}}}$s heat sensitivity underscores the need for tailored processing methods. The findings suggest these probiotics, especially the multifunctional *L. brevis* Cc_3_ holds great promise for developing regionally appropriate functional foods in South Asia. However, further clinical studies are needed to confirm their immunomodulatory properties and safety for human consumption.

## Data Availability

The datasets presented in this study can be found in online repositories. The names of the repository/repositories and accession number(s) can be found in the article/supplementary material.
